# Intervenção Coronária Percutânea Primária Fora do Horário de Expediente: Experiência de uma Década em Centro Cardiovascular de Alto Volume

**DOI:** 10.36660/abc.20240396

**Published:** 2024-10-30

**Authors:** Filipe Cirne, Marcia Moura Schmidt, Cristiano Oliveira Cardoso, Darryl P. Leong, Alexandre Schaan de Quadros

**Affiliations:** 1 Instituto de Cardiologia Programa de Pós-Graduação em Ciências da Saúde Porto Alegre RS Brasil Instituto de Cardiologia – Programa de Pós-Graduação em Ciências da Saúde, Porto Alegre, RS – Brasil; 2 McMaster University Department of Medicine Hamiton Ontario Canadá McMaster University – Department of Medicine, Hamiton, Ontario – Canadá; 3 Instituto de Cardiologia Porto Alegre RS Brasil Instituto de Cardiologia – Hemodinâmica, Porto Alegre, RS – Brasil

**Keywords:** Intervenção Coronária Percutânea Primária, Eventos Cardiovasculares Adversos Maiores, Infarto Agudo do Miocárdio com Supradesnivelamento do Segmento ST

## Abstract

**Fundamento:**

O impacto de se realizar uma intervenção coronária percutânea primária (ICPp) em horário fora do expediente sobre desfechos clínicos não está bem estabelecido.

**Objetivo:**

Comparar as características e a ocorrência de eventos cardiovasculares adversos maiores (MACE) de ICPp realizada fora do horário de expediente versus ICPp realizada em horário de expediente em um centro de cardiologia de alto volume.

**Métodos:**

Estudo prospectivo do tipo coorte de pacientes submetidos à ICPp por Infarto Agudo do Miocárdio com Supradesnivelamento do Segmento ST (IAMCSST) entre 2009 e 2019. Definimos horário fora do expediente como dias de trabalho entre 8pm e 7h59am, além de finais de semana e feriados. Comparamos pacientes tratados em horário de expediente e fora do horário do expediente quanto as características basais e eventos em um ano.

**Resultados:**

Um total de 2560 pacientes foram tratados fora do horário de expediente e 1876 pacientes tratados em horário de expediente. Os grupos foram similares para a maioria das características basais. Uma carga de trombo mais alta foi observada em pacientes tratados fora do horário de expediente (50% x 45%; p < 0,01), e nesse grupo o acesso radial foi o mais frequentemente utilizado (62% x 58%; p = 0,01). O sucesso do procedimento não foi estatisticamente diferente entre os grupos (95,7% x 96,4%; p = 0,21). As taxas de MACE foram mais altas em pacientes tratados fora do horário de expediente em 30 dias (10,2% x 8,5%; p = 0,04) e em um ano de seguimento (15,4% x 13,1%; p = 0,03), devido às taxas mais elevadas de morte em 30 dias (7,8% x 6,1%; p = 0,03) e em um ano de seguimento (11,1% x 9,0%; p = 0,02).

**Conclusão:**

Em um centro de cardiologia de alto volume, as características clínicas, os tempos porta-balão, o sucesso das ICPps e as taxas de complicação foram similares entre pacientes com IAMCSST tratados em horário de expediente e pacientes tratados fora do horário de expediente. Contudo, os pacientes tratados fora do horário de expediente apresentaram taxas mais altas de MACE e de mortalidade, apesar de taxas similares de infarto do miocárdio e acidente vascular cerebral.

## Graphical abstract

**Figura Central:**
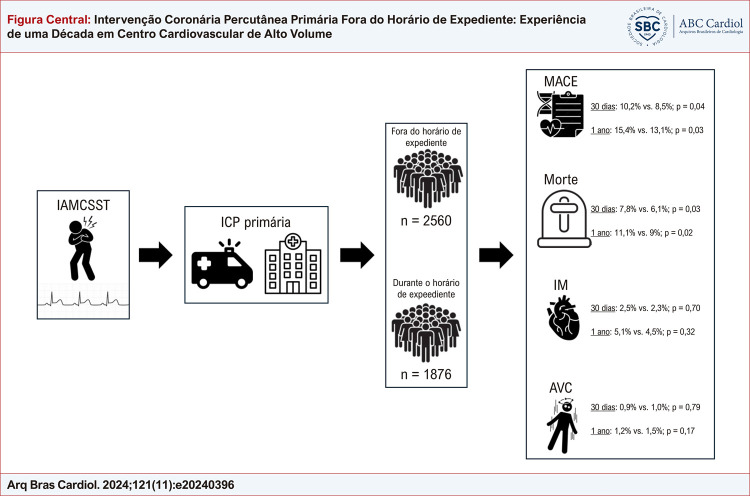
: Intervenção Coronária Percutânea Primária Fora do Horário de Expediente: Experiência de uma Década em Centro Cardiovascular de Alto Volume

## Introdução

No contexto do infarto agudo do miocárdio com supradesnivelamento do segmento ST (IAMCSST), embora superior à trombólise, a intervenção coronária percutânea primária (ICPp) depende de uma infraestrutura mais complexa, incluindo um laboratório de cateterismo e de uma equipe multidisciplinar de suporte. Uma vez que muitos eventos de IAMCSST ocorrem durante a noite e nos finais de semana, isto é, em período fora do expediente de trabalho, esses serviços devem funcionar 24 horas por dia, sete dias da semana. O desempenho das ICPps realizadas em horários fora do expediente parece estar associado com piores desfechos do IAMCSST, com possíveis razões incluindo demora no tempo entre o início de sintomas e a internação hospitalar e no tempo porta-balão, variações fisiológicas circadianas, e padrões distintos de assistência.^1-5^ Em um estudo, Henriques et al.^3^ mostraram que, apesar da ausência de diferenças nas características basais clínicas ou demora no tratamento, os pacientes tratados em períodos fora do horário de expediente apresentaram taxas mais altas de falha na angioplastia (6,9% vs. 3,8%; p<0,01) bem como uma mortalidade mais alta em 30 dias (4,2% vs 1,9%, p<0,01).^1-5^

Por outro lado, não foram observadas diferenças em desfechos clínicos entre ICPp realizada no horário e fora do horário de expediente em estudos mais recentes.^6-10^ Um estudo francês relatou que não houve diferença entre ICPp realizada durante ou fora do horário de expediente em relação à mortalidade hospitalar (8,1% vs. 7,0%; p = 0,49) ou mortalidade em um ano (11,0% vs. 11,1%; p = 0,89) respectivamente. Vale ressaltar que as características basais eram bem equilibradas entre os dois grupos, incluindo taxas de parada cardíaca (7,9% vs. 8,8%; p = 0,55) e de choque cardiogênico (12,3% vs. 14,7%; p = 0,16). Além disso, o tempo mediano entre os primeiros sintomas e o contato médico e o tempo mediano entre o contato médico e a inserção do cateter não foram diferentes (120 minutos vs. 126 minutos; p = 0,25 e 90 minutos vs. 93 minutos; p = 0,58; respectivamente), bem como a taxa de acesso radial para o cateterismo (85,6% vs. 87,5%; p = 0,27).^10^ Redes de assistência regionais organizadas, com padronização de rotinas e ampla disponibilidade de centros de ICPp, capazes de realizar esses procedimentos em tempo hábil, 24 horas por dia, sete dias por semana, devem ser considerados para esse efeito similar entre ICPp realizada em horário de expediente e fora de horário de expediente nessas coortes contemporâneas.

Portanto, considerando os resultados conflitantes na literatura sobre o impacto de se submeter à ICPp em horário de expediente *versus* fora do horário de expediente quanto os desfechos clínicos dos pacientes com IAMCSST, nosso objetivo foi avaliar as características clínicas e desfechos clínicos em curto e em longo prazo de uma coorte contemporânea de pacientes com IAMCSST tratados com ICPp fora do horário de expediente em um centro terciário de cardiologia durante um período de dez anos.

## Métodos

### Delineamento do estudo e população

Estudo prospectivo do tipo coorte de pacientes consecutivos com IAMCSST tratados com ICPp no Instituto de Cardiologia do Rio Grande do Sul entre 2009 e 2019. Nossa instituição é um centro acadêmico de referência em cardiologia para o tratamento de condições cardíacas complexas no sul do Brasil, que cobre uma região de aproximadamente 4,5 milhões de pessoas. O laboratório de cateterismo está disponível 24 horas por dia, sete dias da semana, e realiza aproximadamente 3000 ICPps por ano.

Os critérios de inclusão foram IAMCSST com menos de 12 horas desde o início dos sintomas, ou com mais de 12 horas em caso de dor persistente e/ou evidência de isquemia. Excluímos pacientes com idade inferior a 18 anos e aqueles que se recusaram a assinar o termo de consentimento.

Os pacientes foram separados em dois grupos: 1) ICP realizada durante horário de expediente (dias de trabalho regulares, entre 8h e 19h59, e 2) ICP realizada fora do horário de expediente (dias de trabalho regulares entre 20h e 7h59, e fins de semana e feriados).

O protocolo foi aprovado pelo comitê de ética local; todos os pacientes incluídos assinaram um termo de consentimento.

### Procedimento de ICPp

Depois que o diagnóstico de IAMCSST foi confirmado, todos os pacientes receberam 300 mg de ácido acetilsalicílico (AAS) e um inibidor de P2Y12, 600 mg de clopidogrel ou 180 mg de ticagrelor. Os pacientes foram imediatamente transferidos ao laboratório de cateterismo para serem submetidos a um angiograma coronário e a uma ICPp se apropriado. O local de acesso vascular, uso de pré-dilatação, administração inibidores de glicoproteína IIb/IIIa, tromboaspiração e implante de *stent* foram decididos pelo técnico atendente. Todas as ICPs foram realizadas por cardiologistas treinados e experientes, e seguindo diretrizes nacionais e internacionais.^11-14^

### Definições

IAMCSST como um critério de inclusão foi definido como (1) dor ou equivalente em repouso associada com elevação de segmento ST ≥ 1 mm em pelo menos duas derivações contíguas no eletrocardiograma (ECG); ou (2) dor ou equivalente em repouso associada com um bloqueio de ramo esquerdo (BRE) presumidamente novo no ECG. Infarto foi classificado de acordo com a Quarta Definição Universal.^15^

O tempo porta-balão foi definido como o tempo entre a admissão hospitalar e a primeira inflação do balão de angioplastia ou o implante do *stent* (o que ocorreu primeiro). O tempo entre início de sintoma e admissão hospitalar foi definido como o tempo pré-admissão. Uma carga trombótica elevada foi definida como trombos claramente visíveis na angiografia coronária.

De acordo com a classificação TIMI (*Thrombolysis in Myocardial Infarction*) sangramento maior foi definido como sangramento clinicamente evidente com uma queda nos níveis de hemoglobina ≥ 5g/dL, ou hemorragia cerebral, ou sangramento fatal, ou, se relacionado à cirurgia de *bypass* da artéria coronária, sangramento fatal, necessidade de procedimento cirúrgico para interromper a cirurgia, sangramento intracraniano no perioperatório, necessidade de transfusão com cinco ou mais bolsas de hemácias dentro de 48 horas após a cirurgia ou drenagem de mais de dois litros dentro de 24 horas. Sangramento menor foi definido como presença de sangramento clinicamente evidente com uma queda nos níveis de hemoglobina entre 3g/dL e 5g/dL ou hematócrito entre 9% e 15%.^16^ Sangramento “não-TIMI” foi definido como aquele que não preenchia as definições acima, mas requereu cuidado médico, testes laboratoriais ou de imagens, ou cirurgia. Sepse foi definida como a presença de infecção acompanhada por sinais e/ou sintomas. Lesão renal aguda foi definida como um aumento nos níveis de creatinina ≥ 50% a partir dos níveis basais.

### Desfechos

O desfecho primário foi um desfecho composto de eventos cardiovasculares adversos maiores (MACE, do inglês *Major Adverse Cardiac Events*) consistindo em morte, infarto do miocárdio (IM), e acidente vascular cerebral (AVC) em um ano. Desfechos secundários foram MACE em 30 dias, cada componente do desfecho primário em 30 dias e em um ano, trombose de *stent* e necessidade de uma nova ICP. IM foi definido como dor torácica recorrente com nova elevação de biomarcadores séricos, após um declínio inicial da curva natural, com elevação do segmento ST ou novas ondas Q, de acordo com a definição universal de IM.^15^ AVC foi definido como um novo déficit neurológico focal de início repentino, de causa presumidamente cerebrovascular, irreversível (ou resultando em morte) dentro de 24 horas, e não causado por outra causa prontamente identificável. AVC foi classificado como isquêmico ou hemorrágico. Uma nova ICP foi composta da revascularização do vaso alvo e da revascularização do vaso não culpado.

### Análise estatística

Todos os dados foram coletados prospectivamente, inseridos em um banco de dados específico e analisados com o programa *Statistical Package for the Social Sciences* (SPSS version 19.0 for Windows).

Normalidade da distribuição dos dados foi confirmada pelo teste de Kolmogorov-Smirnov test. As variáveis contínuas foram descritas como média ± desvio padrão, ou, se mais apropriado, como mediana ± intervalo interquartil. As variáveis categóricas foram descritas como frequências absolutas e relativas. As variáveis contínuas foram comparadas pelo teste t, e as variáveis categóricas pelo teste do qui-quadrado ou o teste exato de Fisher. A análise do tempo de sobrevida foi realizada pelo método de Kaplan-Meier e os desfechos dos pacientes submetidos à ICP em horário de expediente e em horário fora de expediente foram comparados pelo teste de log-rank. Os preditores de MACE e de morte foram avaliados pelos modelos de risco proporcional de Cox. As variáveis foram incluídas no modelo de regressão se o p valor fosse menor que 0,05, ou por relevância clínica demonstrada em estudos anteriores. Um p <0,05 foi considerado estatisticamente significativo.

## Resultados

Entre dezembro de 2009 e dezembro de 2019, 4436 pacientes consecutivos com IAMCSST foram tratados com ICPp em nosso centro. Um total de 2560 pacientes foram tratados fora do horário de expediente, enquanto 1876 foram tratados em horário de expediente. Em um ano, 31 (1,2%) pacientes atendidos fora do horário de expediente e 24 atendidos no horário de expediente perderam seguimento no estudo. No geral, ambos os grupos apresentaram características clínicas basais similares, com exceção de uma prevalência mais alta de fumantes entre os pacientes submetidos à ICPp em horário fora de expediente (44,7% vs. 39,3%; p < 0,01), e uma taxa mais alta de CABG prévia entre aqueles submetidos à ICPp em horário de expediente (4,0% vs. 2,8%; p = 0,02). Não foram observadas diferenças estatisticamente significativas entre os grupos quanto à idade, sexo, local do infarto e classificação de Killip (Tabela 1).

**Tabela 1:** – Características basais dos pacientes tratados em horário de expediente e fora do horário de expediente (n = 4436)

Característica	Fora de expediente (n = 2560)	No expediente (n = 1876)	p
Idade (anos)	60 ± 12	61 ± 12	0,18
Sexo masculino	1749 (68,3%)	1318 (70,3%)	0,15
Caucasiano	2023 (86%)	1484 (85,3%)	0,72
**Fatores de risco para DAC**			
Tabagismo	1144 (44,7%)	738 (39,3%)	< 0,01
Hipertensão	1614 (63,3%)	1164 (62,4%)	0,54
DM	655 (25,7%)	489 (26,2%)	0,69
Dislipidemia	807 (31,8%)	546 (29,3%)	0,08
HF de DAC prematura	608 (23,9%)	478 (25,7%)	0,18
**Comorbidades**			
IM prévio	518 (20,8%)	376 (20,5%)	0,83
ICP prévia	422 (17%)	311 (17%)	0,98
CABG prévio	69 (2,8%)	74 (4,0%)	0,02
Insuficiência cardíaca	152 (6,1%)	100 (5,5%)	0,37
AVC/AIT	167 (6,7%)	115 (6,3%)	0,56
Claudicação intermitente	465 (18,8%)	341 (18,7%)	0,94
DRC	63 (2,5%)	42 (2,3%)	0,61
DPOC	123 (4,8%)	103 (5,5%)	0,29
**Tratamento médico nas primeiras 24 horas**			
AAS	2498 (98,6%)	1826 (98%)	0,11
Clopidogrel	2323 (92%)	1689 (91,6%)	0,59
Ticagrelor	30 (1,2%)	25 (1,4%)	0,62
Prasugrel	13 (0,5%)	11 (0,6%)	0,72
Estatina	2106 (83,1%)	1540 (82,7%)	0,69
Betabloqueador	1650 (65,2%)	1215 (65,3%)	0,95
IECA	1556 (61,4%)	1201 (64,4%)	0,05
BRA	113 (4,5%)	73 (3,9%)	0,37
Nitrato parenteral	462 (18,3%)	332 (17,8%)	0,71
Inotrópicos	15 (0,6%)	12 (0,6%)	0,82
Vasopressores	179 (7,1%)	127 (6,8%)	0,74
Tempo sintoma-hospital (minutos)	241 (123;412)	240 (122;400)	0,49
**TIMI RISK score**			0,51
0	1943 (75,8%)	1391 (70%)	
1	267 (10,4%)	205 (10,3%)	
2	129 (5,0%)	109 (5,4%)	
3	258 (10%)	201 (10,1%)	
Pico TnI-us (ng/dL)	3295 (945;7354)	3317 (976;7415)	0,63
IM prévio	1111 (43,5%)	862 (46%)	0,09
Infarto no VE	327 (12,9%)	241 (13%)	0,91
BRE	33 (1,3%)	19 (1%)	0,39
Bloqueio AV completo	97 (3,8%)	58 (3,1%)	0,21
Killip III/IV	187 (7,4%)	129 (6,9%)	0,77

AIT: ataque isquêmico transitório; AAS: ácido acetilsalicílico; AV: atrioventricular; BRA: bloqueador de receptor de angiotensina; BRE: bloqueio de ramo esquerdo; DRC: doença renal crônica; DPOC: doença pulmonar obstrutiva crônica; IECA: inibidor da enzima conversora de angiotensina; IM: infarto do miocárdio; TnI-us: Troponina I ultrassensível.

Com exceção de uma maior carga trombótica no grupo submetido à ICP fora do horário de expediente (49,6% vs. 45,5%; p < 0,01), todas as características angiográficas foram similares, incluindo a extensão da doença arterial coronariana (DAC), a artéria culpada, a complexidade da lesão na artéria culpada, e o grau de fluxo TIMI (Tabela 2).

**Tabela 2:** – Características angiográficas basais dos pacientes tratados fora do horário de expediente (n = 4436)

Característica	Fora do expediente (n = 2560)	No horário de expediente (n = 1876)	p
Extensão da DAC			0,20
Doença de único vaso	1157 (45,8%)	887 (47,9%)	
Doença de dois vasos	853 (33,7%)	605 (32,6%)	
Doença de três vasos	493 (19,5%)	334 (18%)	
Estenose > 50% na artéria principal esquerda	89 (3,5%)	61 (3,3%)	0,68
Vaso culpado			0,31
ADAE	1098 (43,7%)	834 (45,6%)	
Cx	321 (12,8%)	234 (12,8%)	
ACD	1039 (41,4%)	708 (38,8%)	
LM	23 (0,9%)	16 (0,9%)	
AMID/EVS	31 (1,3%)	35 (1,9%)	
Lesão de bifurcação	284 (11,3%)	179 (9,8%)	0,108
Alta carga trombótica	1247 (49,6%)	835 (45,5%)	<0,01
Carga de cálcio moderada/grave	221 (8,8%)	168 (9,2%)	0,66
Grau de fluxo TIMI pré-procedimento			0,43
0	1912 (76,4%)	1363 (74,4%)	
1	246 (9,8%)	192 (10,5%)	
2	114 (4,6%)	98 (5,4%)	
3	229 (9,2%)	178 (9,7%)	

ADAE: artéria descendente anterior esquerda; ACD: artéria coronária direita; AMID: artéria mamária interna direita; Cx: artéria circunflexa; ECS: enxerto de veia safena; TIMI: Thrombolysis in Myocardial Infarction.

O uso de um acesso radial, pós-dilatação após implante de *stent* e administração de inibidores de glicoproteína IIb/IIIa foram mais comuns nos pacientes atendidos fora do horário de expediente. Além disso, o comprimento do *stent* foi significativamente maior nesses pacientes. Não observamos diferença entre os grupos quanto aos tempos porta-balão, uso de trombectomia manual, grau de fluxo TIMI, grau de opacificação do miocárdio após o procedimento, uso de bomba de balão intra-aórtico, sucesso da ICPp e taxas de complicações do procedimento (Tabela 3).

**Tabela 3:** – Aspectos da intervenção coronária percutânea primária de pacientes tratados em horário de expediente e fora do horário de expediente (n = 4436)

Características	Fora do horário de expediente (n = 2560)	No horário de expediente (n = 1876)	p
Tempo porta-balão (minutos)	70 (53;95)	69 (50;95)	0,15
Acesso radial	1544 (62,1%)	1061 (57,6%)	0,01
Pré-dilatação	1684 (68,3%)	1186 (65,9%)	0,09
Trombectomia manual	470 (19,0%)	318 (17,6%)	0,24
**Tipo do *stent***			0,96
Metálico	1729 (75,8%)	1256 (75,9%)	
Farmacológico	551 (24,2%)	399 (24,1%)	
Diâmetro do *stent* (mm)	3,1 ± 1,2	3,1 ± 0,9	0,12
Comprimento do *stent* (mm)	23 ± 7	22 ± 8	<0,01
> 1 *stent*	488 (20,1%)	378 (21,4%)	0,29
Angioplastia *ad-hoc* de vaso não culpado	54 (2,2%)	46 (2,6%)	0,41
Pós-dilatação	813 (33,2%)	484 (27,3%)	<0,01
% estenose residual	3,1 ± 16	3,6 ± 17	0,33
**Grau do fluxo TIMI pós-procedimento**			0,69
0	67 (2,7%)	39 (2,2%)	
1	31 (1,3%)	23 (1,3%)	
2	108 (4,5%)	84 (4,9%)	
3	2262 (91,7%)	1648 (91,9%)	
**Grau de opacificação (*blush*) do miocárdio pós-procedimento**			0,31
0	174 (7,1%)	104 (5,8%)	
1	80 (3,3%)	69 (3,9%)	
2	190 (7,7%)	140 (7,9%)	
3	2016 (82%)	1469 (82,4%)	
Sucesso do procedimento	2356 (95,7%)	1726 (96,4%)	0,21
Inibidores de glicoproteína IIb/IIIa	659 (26,2%)	407 (22,1%)	<0,01
Bomba de balão intra-aórtico	83 (3,3%)	54 (2,9%)	0,49
Marcapasso temporário	107 (4,2%)	88 (4,7%)	0,42
**Complicações do procedimento**			0,97
Dissecção da artéria coronária	59 (2,3%)	43 (2,3%)	
Perfuração da artéria coronária	3 (0,1%)	2 (0,1%)	
Ausência de refluxo	88 (3,5%)	54 (2,9%)	
Oclusão aguda do vaso culpado	13 (0,5%)	9 (0,5%)	
Oclusão do vaso do ramo	26 (1,0%)	27 (1,5%)	
FV/TV	67 (2,7%)	49 (2,7%)	
Bloqueio AV completo	25 (1,0%)	16 (0,9%)	
Acidente Vascular Cerebral	4 (0,2%)	3 (0,2%)	
Morte	9 (0,4%)	9 (0,5%)	

AV: atrioventricular; TIMI: Thrombolysis in Myocardial Infarction; FV: fibrilação ventricular; TV: taquicardia ventricular.

Embora a ocorrência de MACE durante a internação não tenha sido estatisticamente diferente entre os grupos, a ocorrência de MACE em 30 dias (10,2% vs. 8,5%, p = 0,04) e em um ano foi maior (15,4% vs. 13,1%; p = 0,02) nos pacientes tratados em horário fora de expediente que naqueles tratados em horário de expediente (Tabela 4). Os pacientes tratados em horário fora de expediente apresentaram maiores taxas de mortalidade hospitalar (7,9% vs. 6,1%, p = 0,02), em 30 dias (7,8% vs. 6,1%, p = 0,03) e em um ano (11,1% vs. 9%, p = 0,02). IM e AVC não foram estatisticamente diferentes entre os grupos (Figura Central). Os pacientes submetidos à ICPp em horário fora de expediente apresentaram taxas mais altas de ICP em vasos não culpados (13,6% vs. 11,2%; p = 0,04) e uma taxa mais baixa de nova revascularização de vaso alvo (3,0% vs. 3,6%; p = 0,04). Outros desfechos clínicos e complicações hospitalares, tais como sangramento maior e menor, trombose de *stent* e lesão renal aguda foram similares entre os grupos (Tabela S1 no material suplementar).

**Tabela 4:** – Desfechos clínicos dos pacientes tratados em horário de expediente e fora do horário de expediente (n = 4436)

	Fora do horário de expediente (n = 2560)	No horário de expediente (n = 1876)	p
**Durante internação**			
MACE	239 (9,4%)	148 (8%)	0,09
Morte	200 (7,9%)	114 (6,1%)	0,02
IM	39 (1,5%)	29 (1,6%)	0,95
AVC	19 (0,8%)	17 (0,9%)	0,54
Trombose de *stent*	32 (1,3%)	29 (1,6%)	0,40
**30 dias**			
MACE	259 (10,2%)	157 (8,5%)	0,04
Morte	197 (7,8%)	113 (6,1%)	0,03
IM	58 (2,5%)	40 (2,3%)	0,70
AVC	24 (0,9%)	19 (1,0%)	0,79
Nova ICP	381 (16,4%)	265 (15,3%)	0,34
**1 ano**			
MACE	389 (15,4%)	242 (13,1%)	0,03
Morte	280 (11,1%)	166 (9%)	0,02
IM	117 (5,1%)	76 (4,5%)	0,32
AVC	31 (1,2%)	27 (1,5%)	0,50
Nova ICP	452 (20%)	309 (18,3%)	0,17

IM: infarto do miocárdio; AVC: acidente vascular cerebral; MACE: cardiovasculares adversos maiores (major adverse cardiovascular events); ICP: intervenção coronária percutânea.

Os preditores independentes de MACE em um ano foram ICPp realizada em horário fora de expediente, tempo pré-admissão prolongado, pior classificação de Killip, acesso radial, doença de múltiplos vasos, lesão na artéria coronária principal esquerda e grau de fluxo TIMI mais baixo após o procedimento, de acordo com o modelo de riscos proporcionais de Cox (Tabela 5). Tabagismo foi inversamente associado com a ocorrência de MACE em um ano. Os preditores independentes de morte em um ano foram todos os mencionados acima e diabetes mellitus. Além disso, o tabagismo associou-se inversamente com morte em um ano.

**Tabela 5:** – Modelo de riscos proporcionais de Cox para eventos cardiovasculares adversos maiores (MACE) e mortalidade em um ano

	MACE			morte		
Variável	HR	IC95%	p	HR	IC95%	p
Fora do horário de expediente	1,26	1,06-1,49	0,01	1,3	1,08-1,66	<0,01
Idade	1,03	1,02-1,04	<0,01	1,04	1,03-1,05	<0,01
Sexo masculino	0,90	0,75-1,08	0,25	0,83	0,67-1,02	0,08
Tabagismo	0,79	0,63-0,99	0,04	0,73	0,56-0,96	0,02
Diabetes mellitus	1,16	0,97-1,39	0,11	1,28	1,03-1,59	0,03
Tempo sintoma-hospital	1,01	1,0-1,01	<0,01	1,01	1,0-1,01	<0,01
Killip	1,76	1,63-1,9	<0,01	2,07	1,9-2,25	<0,01
Acesso radial	1,65	1,37-1,97	<0,01	1,62	1,3-2,03	<0,01
Número de vasos afetados	1,18	1,05-1,31	<0,01	1,16	1,02-1,33	0,02
Lesão na artéria coronária principal esquerda	1,75	1,27-2,43	<0,01	1,94	1,36-2,77	<0,01
Carga trombótica elevada	1,10	0,93-1,3	0,27	1,02	0,83-1,25	0,86
Grau de fluxo TIMI após o procedimento	0,67	0,60-0,74	<0,01	0,60	0,53-0,67	<0,01

IC: intervalo de confiança; HR: hazards ratio; TIMI: Thrombolysis in Myocardial Infarction.

**Figura 1:**
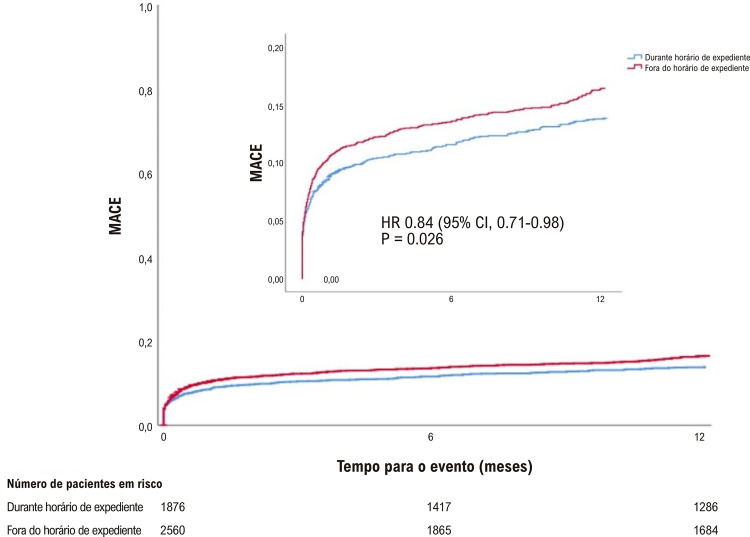
– Estimativa de Kaplan-Meier para eventos cardiovasculares adversos maiores (MACE).

**Figura 2 f03:**
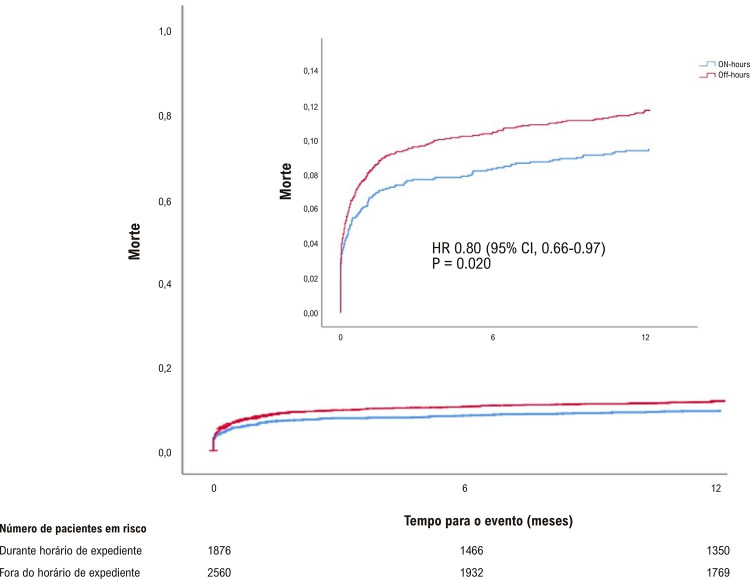
– Estimativa de Kaplan-Meier para mortes.

**Figura 3 f04:**
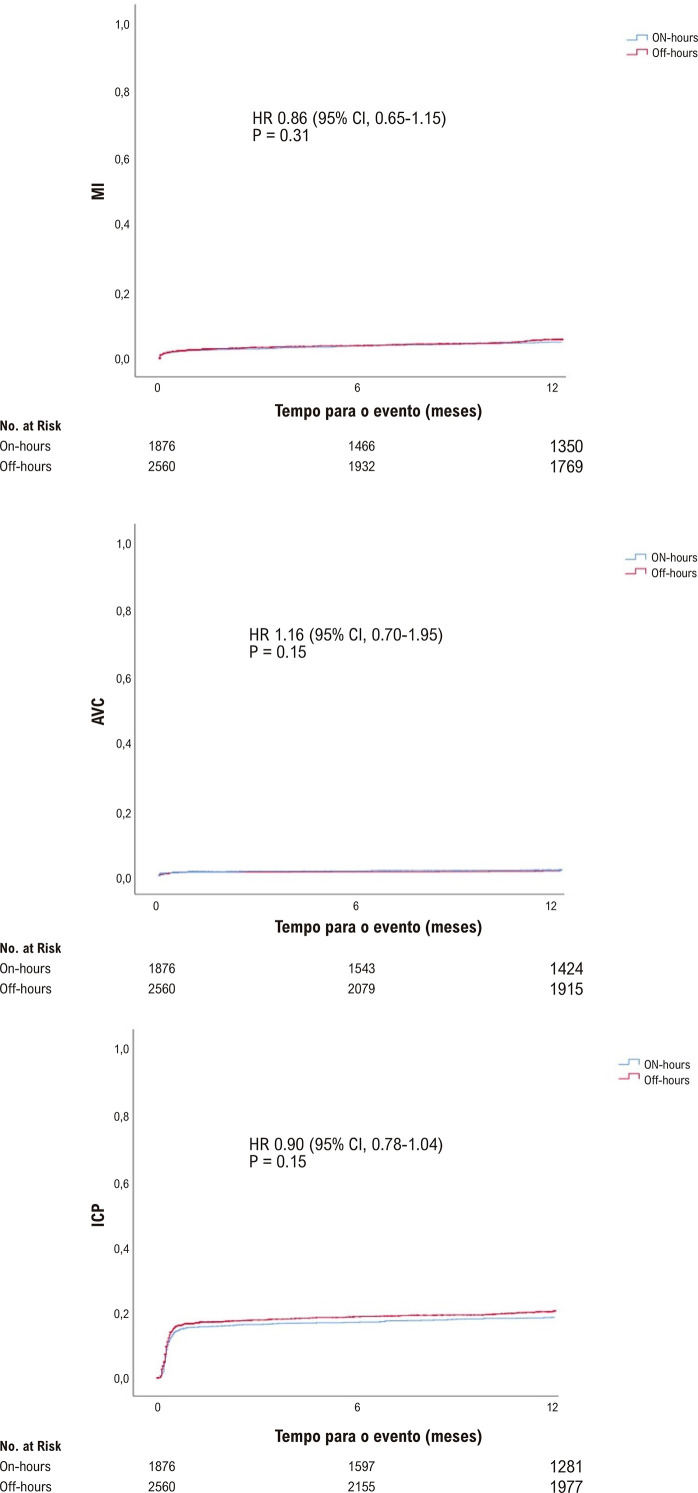
– Estimativa de Kaplan-Meier para infarto do miocárdio, acidente vascular cerebral e intervenção coronária percutânea. IM: infarto do miocárdio; AVC: acidente vascular cerebral; ICP: intervenção coronária percutânea.

As Figuras 1-3 [Fig f03] [Fig f04] representam as curvas de Kaplan-Meier para a comparação entre os grupos de MACE, mortalidade, infarto agudo do miocárdio, AVC e ICP, mostrando taxas mais altas de MACE e morte quando a ICPp foi realizada fora do horário de expediente, com separação precoce das curvas, antes de seis meses de seguimento.

## Discussão

Em uma população de pacientes de um grande centro de referência em cardiologia, pacientes com IAMCSST que chegaram ao hospital fora do horário de expediente não apresentaram maior tempo entre início de sintomas e chegada ao hospital (pré-admissão) nem maior tempo porta-balão, e apresentaram resultados imediatos da ICPp similares em comparação a pacientes tratados durante o horário de expediente. Contudo, as taxas de MACE e de mortalidade em 30 dias e em um ano foram significativamente mais altas em pacientes tratados fora do horário de expediente. No presente estudo, aproximadamente 40% dos pacientes tratados em nosso hospital necessitaram de cuidado em horários fora do expediente, e o alcance de um tempo porta-balão e resultados imediatos da ICPp similares a de pacientes tratados em horário de expediente reinforça a importância de um cuidado estruturado para lidar com pacientes com IAMCSST, de maneira mais homogênea possível, 24 horas por dia, sete dias da semana.

Considerando que o IAMCSST é uma condição sensível ao tempo no que diz respeito aos resultados e desfechos ótimos da revascularização,^17,18^ diferentes diretrizes ao redor do mundo defendem curtos tempos porta-agulha e porta-balão, os quais devem ser inferiores a 30 minutos e 90 minutos, respectivamente.^19-21^ Alguns estudos mais antigos mostraram tempos de isquemia mais longos quando a ICPp foi realizada fora do horário de expediente.^5,22-25^ Com a padronização das práticas ao longo do tempo, tempos similares de isquemia foram alcançados independentemente da localização geográfica, do dia da semana ou do horário do dia, e consequentemente nenhuma diferença nos desfechos clínicos.^8,10,26^ Ainda, não encontramos diferenças significativas no tempo entre pré-admissão nem no tempo porta-balão entre os grupos, apesar de taxas mais elevadas de MACE e morte nos pacientes tratados fora do horário de expediente.

Entre os estudos prospectivos comparando ICPp realizada em horário de expediente e fora de expediente, nosso estudo é um dos maiores estudos publicados, o maior de um país de renda média, e o mais contemporâneo. Em outro estudo recente, publicado em 2019, Lattuca et al.^10^ analisaram dados de 2167 pacientes consecutivos com IAMCSST tratados no Hospital Universitário Pitié-Salpêtrière em Paris. As taxas de mortalidade em um ano foram similares entre os pacientes tratados em horário de expediente e fora do horário de expediente (HR = 0,94, IC95% 0,64-1,37; p = 0,89).^10^ Vale destacar que nosso tamanho amostral foi duas vezes maior que o do estudo de Lattuca et al.,^10^ o que pode ter aumentado nosso poder estatístico e nos possibilitado mostrar uma diferença significativa na mortalidade e nos MACE entre os grupos.

Os principais achados deste estudo – taxas mais altas de MACE e de mortalidade nos pacientes tratados fora do horário de expediente – devem ser analisados diante de algumas características específicas de nosso estudo. Primeiro, os pacientes tratados em ambos os grupos foram similares no geral, com exceção da maior carga trombótica nos pacientes tratados fora do horário de expediente. Esse aspecto está normalmente associado a taxas mais altas de complicações, ausência de refluxo, taxas de sucesso das ICCPs mais baixas e piores desfechos, mas em nosso estudo, os resultados da ICPp foram similares entre os dois grupos. Segundo, os tempos porta-balão foram similares em ambos os grupos, mostrando acesso similar ao cuidado entre pacientes tratados dentro e fora do horário de expediente. Terceiro, o principal fator associado às taxas elevadas de MACE foi uma maior mortalidade no grupo tratado fora do horário de expediente, sem diferenças estatisticamente significativa nas taxas de evento isquêmico. Esses achados sugerem que, apesar de apresentar características clínicas, angiográficas e do procedimento similares a de pacientes tratados em horário de expediente, os indivíduos tratados fora do horário de expediente estão em maior risco. Um aumento na mortalidade total não atribuível a eventos isquêmicos aumenta a possibilidade de diferenças em outras comorbidades não detectadas em nosso banco de dados.

Além disso, encontramos taxas mais altas de tabagismo em pacientes submetidos à ICP realizada fora do horário de expediente, similar a estudos prévios,^7,27,28^ embora a frequência de diagnóstico de doença pulmonar obstrutiva crônica (DPOC) tenha sido similar em ambos os grupos. O uso mais frequente de inibidores de glicoproteína IIb/IIIa por pacientes tratados fora do horário de expediente provavelmente reflete a maior carga trombótica observada nesses pacientes. O uso mais frequente de acesso vascular radial fora do horário de expediente pode refletir operadores mais jovens atuando nesses horários, que foram treinados em um período em que essa via já havia se tornado o padrão-ouro. Finalmente, embora as taxas de trombectomia manual não tenham sido desprezíveis em nosso estudo (cerca de 18%), vale observar que, historicamente, a maioria desses procedimentos foram realizados antes da publicação do estudo TOTAL,^29^ em um período em que a evidência corroborava redução das complicações clínicas por esses procedimentos.^30^

Em nosso modelo de regressão de Cox, mostramos que a associação entre ICPp realizada fora do horário de expediente e taxas mais elevadas de MACE e morte foi independente de fatores de risco conhecidos para eventos nesse contexto. De fato, a maioria das variáveis incluídas no modelo foram preditivas de MACE e morte, com exceção do sexo masculino, carga trombótica elevada, e diabetes. Tabagismo mostrou uma relação inversa com MACE e morte, associação previamente conhecida e demonstrada como o “paradoxo do fumante”.^31,32^ Por fim, é provável que a associação entre acesso radial e um aumento de 65% e 62% no risco de MACE e morte, respectivamente, tenha sido falsa, considerando sua conhecida superioridade sobre o acesso femoral mostrada em estudos randomizados anteriores.^33-35^

Uma limitação deste estudo está relacionada a sua natureza unicêntrica. Contudo, nossa instituição é um centro de referência para ICPp em uma região com 34 cidades e 4,3 milhões de pessoas, com livre acesso por meio do sistema público de saúde no Brasil. O achado de taxas mais altas de morte no grupo submetido à ICPp em horário fora de pico, sem resultados piores do procedimento ou maior ocorrência de eventos isquêmicos levanta a possibilidade de características basais de maior risco nesse grupo, não detectadas por nosso banco de dados. Nesse sentido, a falta de avalição da incidência de insuficiência cardíaca no seguimento, da fração de ejeção do ventrículo esquerdo em médio e longo prazo, das taxas de acompanhamento regular com um cardiologista, da adesão a terapias farmacológicas e não farmacológicas, e de mortes por doenças não cardiovasculares, como o câncer, também pode representar limitações. Fatores como diferenças técnicas entre equipes trabalhando em horário de expediente e fora desse horário, impacto de longas horas de trabalho e privação de sono não parecem justificar as diferenças identificadas, uma vez que as taxas de sucesso foram similares entre os grupos. Ticagrelor e Prasugrel não foram frequentemente utilizados por questões de reembolso relacionadas ao sistema público de saúde no Brasil, o que pode representar uma limitação ao extrapolar nossos resultados a outros países. A maioria dos pacientes foram tratados com *stents* metálicos, também por questões de reembolso no momento dos procedimentos, mas a frequência de seu uso foi similar entre os grupos.

## Conclusão

Neste grande estudo prospectivo comparando ICPp realizada em horário de expediente e fora de horário de expediente em pacientes com IAMCSST, encontramos que ICPp fora do horário de expediente foi associada com taxas mais altas de MACE e morte em um ano, mas o tempo porta-balão, resultados da ICPp, e taxas de IM, AVC e ICP em 30 dias e em um ano foram similares entre os grupos.
