# Supplementary material for: Intervenção Coronária Percutânea Primária Fora do Horário de Expediente: Experiência de uma Década em Centro Cardiovascular de Alto Volume
Source: Arq Bras Cardiol. 2024 Oct 30;121(11):e20240396. [Article in Portuguese] doi: 10.36660/abc.20240396 (PMC11634306; doi:10.36660/abc.20240396)
Supplement: Translation

# Translation: Primary Percutaneous Coronary Intervention during Off-Hours: One-Decade Experience from a High-Volume Cardiovascular Center

**DOI:** 10.36660/abc.20240396i

**Authors:** Filipe Cirne, Marcia Moura Schmidt, Cristiano Oliveira Cardoso, Darryl P. Leong, Alexandre Schaan de Quadros

**Affiliations:** 1 Brazil Instituto de Cardiologia – Programa de Pós-Graduação em Ciências da Saúde, Porto Alegre, RS – Brazil; 2 Canada McMaster University – Department of Medicine, Hamiton, Ontario – Canada; 3 Brazil Instituto de Cardiologia – Hemodinâmica, Porto Alegre, RS – Brazil

**Keywords:** Primary Percutaneous Coronary Intervention, Major Adverse Cardiovascular Events, ST Elevation Myocardial Infarction

## Abstract

**Background:**

The impact of performing a primary percutaneous coronary intervention (pPCI) off-hours on clinical outcomes is not well established.

**Objective:**

Compare characteristics and major adverse cardiovascular events (MACE) of pPCI off-hours versus on-hours in a high-volume cardiology center.

**Methods:**

Prospective cohort of patients who underwent pPCI for ST elevation myocardial infarction (STEMI) from 2009 to 2019. We defined off-hours pPCI as workdays from 8pm to 7:59 am as well as weekends and holidays. We compared patients treated on- and off-hours as to baseline characteristics and 1-year events.

**Results:**

A total of 2,560 patients were treated off-hours and 1,876 patients treated on-hours. The groups were similar for most of the baseline characteristics. A higher thrombus burden was seen in patients treated off-hours (50% x 45%; p < 0.01), and in this group the radial access was more frequently used (62% x 58%; p = 0.01). Procedural success was not statistically different between the groups (95.7% x 96.4%; p = 0.21). MACE rates were higher in patients treated off-hours at 30 days (10.2% x 8.5%; p = 0.04) and at one year of follow-up (15.4% x 13.1%; p = 0.03), driven by higher death rates at 30 days (7.8% x 6.1%; p = 0.03) and at 1 year follow-up (11.1% x 9.0%; p = 0.02).

**Conclusion:**

In a high-volume cardiology center, clinical characteristics, door-to-balloon times, procedural pPCI success and complication rates of STEMI patients treated on and off-hours were similar. However, patients treated off-hours presented higher MACE and mortality rates, in spite of similar MI and stroke rates.

## Introduction

In the setting of ST elevation myocardial infarction (STEMI), while superior to thrombolysis, primary percutaneous coronary intervention (pPCI) relies on a more complex infrastructure including a catheterization laboratory, and a multidisciplinary team to support it. As many STEMI events occur at night and during weekends, i.e., during “off-hours” periods, these services must function 24 hours a day, seven days a week. It has been suggested that the performance of pPCI during off-hours periods could be associated with worse STEMI outcomes, with possible underlying reasons including differences in pre-hospital delay and door-to-balloon times, physiological circadian variations and distinct patterns of care.^1-5^ Importantly, in one study, Henriques et al.^3^ showed that, despite no difference in baseline clinical characteristics or treatment delays, patients treated off-hours had higher rates of angioplasty failure (6.9% vs. 3.8%, p < 0.01) as well as a higher thirty-day mortality (4.2% vs 1.9%, p < 0.01).^1-5^

On the other hand, no differences in clinical outcomes between pPCI performed on- and off-hours were seen in more recent reports.^6-10^ A French study showed no association between on- versus off-hours pPCI regarding in-hospital (8.1% vs. 7.0%; p = 0.49) or 1-year mortality (11.0% vs. 11.1%; p = 0.89), respectively. Of note, baseline characteristics were well-balanced between the two groups, including rates of cardiac arrest (7.9% vs. 8.8%; p = 0.55) and cardiogenic shock (12.3% vs. 14.7%; p = 0.16). Additionally, median symptom-to-first medical contact time and median first medical contact-to-sheath insertion time were not different (120 min vs. 126 min; p = 0.25 and 90 min vs. 93 min; p = 0.58, respectively), as well as the rate of radial access for catheterization (85.6% vs. 87.5%; p = 0.27).^10^ Organized and regionalized STEMI care networks, with standardization of routines and broad availability of pPCI centers, capable to perform these procedures in a timely manner 24h a day, seven days a week, are to be considered for the similar effectiveness of pPCI on- versus off-hours in these contemporary cohorts.

Therefore, considering the conflicting results in the literature regarding the impact of undergoing pPCI on- versus off-hours on the clinical outcomes of patients with STEMI, our objective was to assess the clinical characteristics as well as short and mid-term clinical outcomes of a contemporary cohort of patients with STEMI treated with pPCI off-hours at a tertiary cardiology center over a 10-year period.

## Methods

### Study design and population

Prospective cohort study of consecutive patients with STEMI treated with pPCI at the Instituto de Cardiologia do Rio Grande do Sul between 2009 and 2019. Our institution is an academic cardiac referral center for treatment of complex cardiac conditions in south Brazil, covering a region with approximately 4.5 million people. The cardiac catheterization laboratory is available 24/7, performing approximately 3000 PCIs per year.

The inclusion criteria were STEMI with less than 12h since the onset of symptoms, or with more than 12h if persistent pain and/or evidence of ischemia were present. We excluded patients under 18 years of age and those who refused to sign informed consent.

Patients were divided into two groups: 1) PCI on-hours, workdays from 8am to 7:59 pm, and 2) PCI off-hours, workdays from 8pm to 7:59 am as well as weekends and holidays.

The protocol was approved by the local ethics committee; all included patients signed the informed consent form.

### Primary PCI procedure

After the diagnosis of STEMI was confirmed, all patients received acetylsalicylic acid (ASA) 300mg and a P2Y12 inhibitor, clopidogrel 600mg or ticagrelor 180 mg. Patients were immediately transferred to the catheterization laboratory to undergo a coronary angiogram and pPCI if appropriate. Vascular access site, use of pre-dilatation, administration of glycoprotein IIb/IIIa inhibitors, tromboaspiration and stent implantation were decided by the attending technician. All pPCIs were performed by trained and experienced interventional cardiologists, and according to national and international guidelines.^11-14^

### Definitions

STEMI as an inclusion criterion was defined as: (1) resting pain or equivalent associated with ST segment elevation ≥1 mm in at least two contiguous leads on the electrocardiogram (ECG); or (2) resting pain or equivalent associated with a presumably new left bundle branch block (LBBB) on ECG. Infarct was classified according to the Fourth Universal Definition.^15^

Door-to-balloon time was determined as the time between the hospital admission and either the first angioplasty balloon insufflation or stent implantation (whatever came first). The time between symptom onset and hospital admission was defined as pre-hospital delay. High thrombotic burden was defined as clearly visible thrombus on coronary angiography.

According to the TIMI (Thrombolysis in Myocardial Infarction) classification, major bleeding was defined as clinically evident bleeding with a drop in hemoglobin levels ≥5g/dl, or cerebral hemorrhage, or fatal bleeding, or, if related to coronary artery bypass graft (CABG) surgery, fatal bleeding, need for surgical procedure to stop the bleeding, perioperative intracranial bleeding, need for transfusion with ≥5 units of packed red blood cells within 48h postoperative or drainage of more than two liters within 24 hours. Minor bleeding was defined as the presence of clinically evident bleeding with a drop in hemoglobin levels between 3g/dl and 5g/dl or hematocrit between 9% and 15%.^16^ Non-TIMI bleeding was defined as the one that did not meet the above definitions, but required medical care, laboratory/imaging tests or surgery. Sepsis was defined as the presence of infection accompanied by systemic signs and/or symptoms. Acute kidney injury was defined as an increase in creatinine levels ≥50% from patient basal levels.

### Study endpoints

The primary endpoint was a composite of major adverse cardiovascular events (MACE), consisting of death, myocardial infarction (MI) and stroke at one year. Secondary outcomes were MACE at 30-days, each component of the primary outcome at 30-days and 1-year, stent thrombosis and the need for a new PCI. MI was defined as recurrent chest pain with new elevation of serum biomarkers, after the initial decline of the natural curve, with ST-segment elevation or new Q waves, according to the universal definition of MI.^15^ Stroke was defined as a new, sudden-onset focal neurological deficit, of presumably cerebrovascular cause, irreversible (or resulting in death) within 24 hours, and not caused by another readily identifiable cause. Stroke was classified as ischemic or hemorrhagic. A new PCI was composed of both target vessel revascularization and non-culprit vessel revascularization.

### Statistical analysis

All the data were collected prospectively, entered in a dedicated database and analyzed with the statistical software Statistical Package for the Social Sciences (SPSS version 19.0 for Windows).

Normality of data distribution was confirmed by the Kolmogorov-Smirnov test. Continuous variables were reported as mean ± standard deviation or, if more appropriate, as median ± interquartile range. Categorical variables were reported as absolute and relative frequencies. Continuous variables were compared by t test, and the chi-square test or Fisher’s exact test was used for categorical variables. Analysis of survival time was done with the Kaplan-Meier method and the outcomes of patients undergoing PCI on- versus off-hours compared by the log rank test. The predictors of MACE and death were assessed by Cox proportional hazards models. Variables were included in the regression model if p value was less than 0.05, or by clinical importance demonstrated in prior studies. A p value < 0.05 was considered as statistically significant.

## Results

Between December 2009 and December 2019, 4,436 consecutive patients with STEMI were treated with pPCI at our center. A total of 2,560 patients were treated off-hours, while 1876 were treated on-hours. At 1 year, 31 (1.2%) patients in the off-hours group and 24 (1.2%) in the on-hours group were lost to follow-up. Overall, both groups had similar clinical characteristics at baseline, except for a higher prevalence of smokers in the off-hours group (44.7% vs. 39.3%; p < 0.01), and a higher rate of previous CABG in the on-hours group (4.0% vs. 2.8%; p = 0.02). No statistical differences were found between the groups with regards to age, sex, infarct location and Killip classification, as shown in Table 1.

**Table 1:** – Baseline clinical characteristics of patients treated on- vs off-hours (n = 4436)

Characteristic	Off-hours (n = 2560)	On-hours (n = 1876)	p
Age (years)	60 ± 12	61 ± 12	0.18
Male sex	1749 (68.3%)	1318 (70.3%)	0.15
Caucasian	2023 (86%)	1484 (85.3%)	0.72
**CAD risk factors**			
Smoking	1144 (44.7%)	738 (39.3%)	< 0.01
Hypertension	1614 (63.3%)	1164 (62.4%)	0.54
DM	655 (25.7%)	489 (26.2%)	0.69
Dyslipidemia	807 (31.8%)	546 (29.3%)	0.08
FH of premature CAD	608 (23.9%)	478 (25.7%)	0.18
**Comorbidities**			
Previous MI	518 (20.8%)	376 (20.5%)	0.83
Previous PCI	422 (17%)	311 (17%)	0.98
Previous CABG	69 (2.8%)	74 (4.0%)	0.02
Heart failure	152 (6.1%)	100 (5.5%)	0.37
Stroke/TIA	167 (6.7%)	115 (6.3%)	0.56
Intermittent claudication	465 (18.8%)	341 (18.7%)	0.94
CKD	63 (2.5%)	42 (2.3%)	0.61
COPD	123 (4.8%)	103 (5.5%)	0.29
**Medical treatment in the first 24 hours**			
ASA	2498 (98.6%)	1826 (98%)	0.11
Clopidogrel	2323 (92%)	1689 (91.6%)	0.59
Ticagrelor	30 (1.2%)	25 (1.4%)	0.62
Prasugrel	13 (0.5%)	11 (0.6%)	0.72
Statin	2106 (83.1%)	1540 (82.7%)	0.69
Beta-blocker	1650 (65.2%)	1215 (65.3%)	0.95
ACEI	1556 (61.4%)	1201 (64.4%)	0.05
ARB	113 (4.5%)	73 (3.9%)	0.37
Parenteral nitrates	462 (18.3%)	332 (17.8%)	0.71
Inotropes	15 (0.6%)	12 (0.6%)	0.82
Vasopressors	179 (7.1%)	127 (6.8%)	0.74
Pre-hospital delay (minutes)	241 (123;412)	240 (122;400)	0.49
**TIMI RISK score**			0.51
0	1943 (75,8%)	1391 (70%)	
1	267 (10,4%)	205 (10,3%)	
2	129 (5,0%)	109 (5,4%)	
3	258 (10%)	201 (10,1%)	
Hs-TnI peak (ng/dL)	3295 (945;7354)	3317 (976;7415)	0.63
Anterior MI	1111 (43.5%)	862 (46%)	0.09
RV infarction	327 (12.9%)	241 (13%)	0.91
LBBB	33 (1.3%)	19 (1%)	0.39
Complete AV block	97 (3.8%)	58 (3.1%)	0.21
Killip III/IV	187 (7.4%)	129 (6.9%)	0.77

ACEI: angiotensin-converting enzyme inhibitors; ARB: angiotensin receptor blocker; ASA: acetylsalycilic acid; AV: atrioventricular; CABG: coronary artery bypass grafting; CAD: coronary artery disease; CKD: chronic kidney disease; COPD: chronic obstructive pulmonary disease; DM: diabetes mellitus; FH: family history; Hs-TnI: high-sensitive Troponin I; LBBB: left bundle branch block; MI: myocardial infarction; PCI: percutaneous coronary intervention; RV: right ventricular; TIA: transient ischemic attack.

Apart from a higher thrombotic burden in the off-hours group (49.6% vs 45.5%; p < 0.01), all other angiographic features were similar, including coronary artery disease (CAD) extension, culprit vessel, culprit lesion complexity and TIMI grade flow classification (Table 2).

**Table 2:** – Baseline angiographic features of patients treated on- vs off-hours (n = 4436)

Characteristic	Off-hours (n = 2560)	On-hours (n = 1876)	p
CAD extension			0.20
1 vessel disease	1157 (45.8%)	887 (47.9%)	
2 vessel disease	853 (33.7%)	605 (32.6%)	
3 vessel disease	493 (19.5%)	334 (18%)	
Left main > 50% stenosis	89 (3.5%)	61 (3.3%)	0.68
Culprit vessel			0.31
LAD	1098 (43.7%)	834 (45.6%)	
Cx	321 (12.8%)	234 (12.8%)	
RCA	1039 (41.4%)	708 (38.8%)	
LM	23 (0.9%)	16 (0.9%)	
LIMA/SVG	31 (1.3%)	35 (1.9%)	
Bifurcation lesion	284 (11.3%)	179 (9.8%)	0.108
Heavy thrombotic burden	1247 (49.6%)	835 (45.5%)	<0.01
Moderate/severe calcium burden	221 (8.8%)	168 (9.2%)	0.66
TIMI grade flow pre-procedure			0.43
0	1912 (76.4%)	1363 (74.4%)	
1	246 (9.8%)	192 (10.5%)	
2	114 (4.6%)	98 (5.4%)	
3	229 (9.2%)	178 (9.7%)	

CAD: coronary artery disease; Cx: circumflex artery; LAD: left anterior descending artery; LIMA: left internal mammary artery; RCA: right coronary artery; SVG: saphenous vein graft; TIMI: Thrombolysis in Myocardial Infarction.

The use of a radial access, post-dilatation after stent implantation and administration of glycoprotein IIb/IIIa inhibitors were more common in the off-hours group. Moreover, the stent length was significantly longer in the off-hours group. We observed no between group differences concerning door-to-balloon times, utilization of manual thrombectomy, TIMI grade flow and myocardial blush grade after the procedure, intra-aortic balloon pump (IABP) use, pPCI success and procedural complication rates (Table 3).

**Table 3:** – Primary percutaneous coronary intervention procedural aspects of patients treated on- vs. off-hours (n = 4436)

Characteristic	Off-hours (n = 2560)	On-hours (n = 1876)	p
Door-to-balloon time (minutes)	70 (53;95)	69 (50;95)	0.15
Radial access	1544 (62.1%)	1061 (57.6%)	0.01
Pre-dilatation	1684 (68.3%)	1186 (65.9%)	0.09
Manual thrombectomy	470 (19.0%)	318 (17.6%)	0.24
**Stent type**			0.96
BMS	1729 (75.8%)	1256 (75.9%)	
DES	551 (24.2%)	399 (24.1%)	
Stent diameter (mm)	3.1 ± 1.2	3.1 ± 0.9	0.12
Stent length (mm)	23 ± 7	22 ± 8	<0.01
> 1 stent	488 (20.1%)	378 (21.4%)	0.29
Non-culprit vessel *ad-hoc* angioplasty	54 (2.2%)	46 (2.6%)	0.41
Post-dilatation	813 (33.2%)	484 (27.3%)	<0.01
% residual stenosis	3.1 ± 16	3.6 ± 17	0.33
**TIMI grade flow post-procedure**			0.69
0	67 (2.7%)	39 (2.2%)	
1	31 (1.3%)	23 (1.3%)	
2	108 (4.5%)	84 (4.9%)	
3	2262 (91.7%)	1648 (91.9%)	
**Blush grade post-procedure**			0.31
0	174 (7.1%)	104 (5.8%)	
1	80 (3.3%)	69 (3.9%)	
2	190 (7.7%)	140 (7.9%)	
3	2016 (82%)	1469 (82.4%)	
Procedural success	2356 (95.7%)	1726 (96.4%)	0.21
Glycoprotein IIb/IIIa inhibitors	659 (26.2%)	407 (22.1%)	<0.01
IABP	83 (3.3%)	54 (2.9%)	0.49
Transient PM	107 (4.2%)	88 (4.7%)	0.42
**Procedural complications**			0.97
Coronary dissection	59 (2.3%)	43 (2.3%)	
Coronary perforation	3 (0.1%)	2 (0.1%)	
No-reflow	88 (3.5%)	54 (2.9%)	
Culprit vessel acute occlusion	13 (0.5%)	9 (0.5%)	
Branch vessel occlusion	26 (1.0%)	27 (1.5%)	
VF/VT	67 (2.7%)	49 (2.7%)	
Complete AV block	25 (1.0%)	16 (0.9%)	
Stroke	4 (0.2%)	3 (0.2%)	
Death	9 (0.4%)	9 (0.5%)	

AV: atrioventricular; BMS: bare metal stent; DES: drug eluting stent; IABP: intra-aortic balloon pump; PCI: percutaneous coronary intervention; PM: pacemaker; TIMI: Thrombolysis in Myocardial Infarction classification; VF: ventricular fibrillation; VT: ventricular tachycardia.

Although in-hospital MACE was not statistically different between groups, but MACE at 30-days (10.2% vs 8.5%, p = 0.04) and at one-year (15.4% vs 13.1%; p = 0.02) were higher in patients treated off- vs on-hours (Table 4). Patients treated off-hours presented higher death rates during hospitalization (7.9% vs 6.1%, p = 0.02), at 30 days (7.8% vs 6.1%, p = 0.03) and at one year (11.1% vs 9%, p = 0.02). MI and stroke were not statistically different between groups (central illustration). The off-hours group had higher rates of non-culprit vessel PCI (13.6% vs. 11.2%; p = 0.04) and lower rate of a new target vessel revascularization (3.0% vs 3.6%; p = 0.04). Other clinical outcomes and in-hospital complications, like major and minor bleeding, stent thrombosis and acute kidney injury were similar between the groups (Table S1 in the supplementary appendix).

**Table 4:** – Clinical outcomes of patients treated on- vs off-hours (n = 4436)

	Off-hours (n = 2560)	On-hours (n = 1876)	p
**In-hospital**			
MACE	239 (9.4%)	148 (8%)	0.09
Death	200 (7.9%)	114 (6.1%)	0.02
MI	39 (1.5%)	29 (1.6%)	0.95
Stroke	19 (0.8%)	17 (0.9%)	0.54
Stent thrombosis	32 (1.3%)	29 (1.6%)	0.40
**30 days**			
MACE	259 (10.2%)	157 (8.5%)	0.04
Death	197 (7.8%)	113 (6.1%)	0.03
MI	58 (2.5%)	40 (2.3%)	0.70
Stroke	24 (0.9%)	19 (1.0%)	0.79
New PCI	381 (16.4%)	265 (15.3%)	0.34
**1 year**			
MACE	389 (15.4%)	242 (13.1%)	0.03
Death	280 (11.1%)	166 (9%)	0.02
MI	117 (5.1%)	76 (4.5%)	0.32
Stroke	31 (1.2%)	27 (1.5%)	0.50
New PCI	452 (20%)	309 (18.3%)	0.17

MI: myocardial infarction; MACE: major adverse cardiovascular events; PCI; percutaneous coronary intervention.

The independent predictors of MACE at one-year were off-hours pPCI, pre-hospital delay, worse Killip classification, radial access, multivessel disease, left main lesion and lower TIMI grade flow following the procedure, according to the Cox proportional hazards model (Table 5). Smoking was inversely associated with the occurrence of MACE at one-year. The independent predictors of death at one-year were all those mentioned above and diabetes mellitus. Smoking was also inversely associated with one-year death.

**Table 5:** – Cox proportional hazards multivariate regression analysis for MACE and 1-year death

Variable	MACE			Death		
	HR	95% CI	p	HR	95% CI	p
Off-hours	1.26	1.06-1.49	0.01	1.3	1.08-1.66	<0.01
Age	1.03	1.02-1.04	<0.01	1.04	1.03-1.05	<0.01
Male sex	0.90	0.75-1.08	0.25	0.83	0.67-1.02	0.08
Smoking habit	0.79	0.63-0.99	0.04	0.73	0.56-0.96	0.02
Diabetes mellitus	1.16	0.97-1.39	0.11	1.28	1.03-1.59	0.03
Pre-hospital delay	1.01	1.0-1.01	<0.01	1.01	1.0-1.01	<0.01
Killip	1.76	1.63-1.9	<0.01	2.07	1.9-2.25	<0.01
Radial access	1.65	1.37-1.97	<0.01	1.62	1.3-2.03	<0.01
Number of diseased vessels	1.18	1.05-1.31	<0.01	1.16	1.02-1.33	0.02
Left main lesion	1.75	1.27-2.43	<0.01	1.94	1.36-2.77	<0.01
Heavy thrombotic burden	1.10	0.93-1.3	0.27	1.02	0.83-1.25	0.86
TIMI grade flow post-procedure	0.67	0.60-0.74	<0.01	0.60	0.53-0.67	<0.01

CI: confidence interval; HR: hazards ratio; MACE: major adverse cardiovascular events; TIMI: Thrombolysis in Myocardial Infarction score.

Figures 1 to 3 [Fig f03001] [Fig f04001] represent the Kaplan-Meier curves for the comparison between groups of MACE, death, acute myocardial infarction, stroke and PCI, showing higher rates of MACE and death when the pPCI was done off-hours, with early separation of the curves, before six months of follow-up.

**Figure 1:** – Kaplan-Meier estimate for MACE (major adverse cardiovascular events).

**Figure 2 f03001:** – Kaplan-Meier estimate for death.

**Figure 3 f04001:** – Kaplan-Meier estimates for myocardial infarction, stroke and percutaneous coronary intervention. MI: myocardial infarction; PCI: percutaneous coronary intervention.

## Discussion

In a population of patients from a high-volume cardiac referral center, STEMI patients arriving at the hospital off-hours did not show significantly longer pre-hospital or door-to-balloon times and showed similar immediate pPCI results when compared to those treated during on-hours. However, 30-day and one-year MACE and death rates were significantly higher in patients treated in the off-hours period. In the present study, approximately 40% of the patients treated in our hospital needed care in the off-hours period, and the achievement of similar door-to-balloon times and immediate pPCI results than those treated on-hours reinforce the importance of providing structured care to deal with STEMI patients as homogeneously as possible on a 24/7 basis.

Acknowledging that STEMI is a time-sensitive condition with regards to optimal revascularization results and outcomes,^17,18^ different guidelines worldwide advocate for short door-to-needle and door-to-balloon times, recommending that they should be ideally less than 30 minutes and 90 minutes, respectively.^19-21^ Some older studies showed longer ischemic times when pPCI was done off-hours.^5,22-25^ With standardization of practices over time, similar ischemic times were achieved regardless of geographic location, day of the week or time of the day, and consequently no differences in clinical outcomes.^8,10,26^ Similarly, we found no statistical differences in pre-hospital delay and door-to-balloon times between the groups, albeit higher rates of MACE and death were seen in patients treated off-hours.

Among prospective studies comparing pPCI on versus off-hours, our study is one of the largest published, the largest from a middle-income country, and the most contemporary study. In another recent study, published in 2019, Lattuca et al.^10^ analyzed data of 2,167 consecutive patients with STEMI treated at the University Hospital Pitié-Salpêtrière in Paris. Mortality rates in 1 year were similar for patients treated on- versus off-hours (HR = 0.94, 95% CI 0.64-1.37; p = 0.89).^10^ It is worth highlighting that our sample size was twofold when compared to the study by Lattuca et al.,^10^ which may have increased our statistical power and enabled us to show a significant difference in death and MACE between groups.

The main findings of our study, higher MACE and death rates in patients treated off-hours, should be viewed in the light of some specific characteristics of our study. Firstly, patients treated in both groups were generally similar, but off-hours patients had a higher thrombotic burden. This aspect is generally associated with higher complication rates, no-reflow, lower pPCI success rates and worse outcomes, but in our study pPCI results were similar between both groups. Secondly, door-to-balloon times were similar in both groups, which shows similar access to care between patients treated off- and on-hours. Thirdly, the main driver of increased MACE rates was a higher mortality in the off-hours group, with no statistically significant differences in the rates of ischemic events. These findings may suggest that, despite having similar clinical, angiographic and procedural characteristics to patients treated on-hours, individuals treated off-hours are at higher risk. An increase in total mortality not attributable to higher ischemic events raises the possibility of differences in other comorbid conditions not captured in our database.

We found higher rates of smoking in patients undergoing primary PCI off-hours, like several previous studies also reported,^7,27,28^ but the frequency of a chronic obstructive pulmonary disease (COPD) diagnosis was similar in both groups. The more frequent use of glycoprotein IIb/IIIa inhibitors in patients treated off-hours probably reflects the higher thrombotic burden seen in these patients. The fact that radial vascular access was more often used off-hours may reflect younger operators working in those hours, since they were trained in a period when this route has already become the gold standard of care. Finally, although manual thrombectomy rates were not negligible in our study, around 18%, it is important to note that historically most of these procedures were performed before the publication of the TOTAL study,^29^ in a time when the evidence was supportive of those procedures reducing clinical complications.^30^

In our Cox regression model, we show that the association of off-hours pPCI with higher MACE and death was independent of known risk factors for events in these setting. Indeed, most variables included in the model were predictive of MACE and death, except for male sex, high thrombotic burden and diabetes. Smoking habit showed an inverse relationship with MACE and death, an association already demonstrated and known as the “Smoker´s paradox”.^31,32^ Lastly, it is likely that the association of radial access with a 65% and 62% increase in the risk for MACE and death, respectively, was spurious, considering its known superiority over femoral access from prior randomizes studies.^33-35^

A limitation of this study relates to its single center nature. However, our institution is a referral center for pPCI in a region with 34 cities and 4.3 million people, with free access through the public health system in Brazil. The finding of higher death rates in the off-hours group without worse pPCI results or higher ischemic events raises the possibility of higher-risk baseline features in this group not captured by our database. In this regard, the lack of assessment of the incidence of heart failure in the follow-up, left ventricle ejection fraction in the mid and long term, rates of regular follow-up with a cardiologist, adherence to pharmacological and non-pharmacological therapies, and non-CVD death, including cancer-related death, may also represent limitations. Factors like technical differences among teams working on- and off-hours, impact of longer working hours and sleep deprivation do not seem to justify the differences found as the procedure success rates were similar between groups. Ticagrelor and prasugrel were not used frequently due to reimbursement issues related to the public health system in Brazil, which may represent a limitation when extrapolating our findings to other countries. Most patients were treated with bare metal stents, also due to reimbursement issues at the time of the procedures, but the frequency of its use was similar in both groups.

## Conclusion

In this large contemporary prospective study comparing primary PCI done on- versus off-hours in patients with STEMI, we found that off-hours pPCI was associated with higher rates of MACE and death at 1 year, but door-to-balloon time, pPCI results and rates of MI, stroke and PCI at 30 days and one year were similar between the groups.
